# 4-Bromo-1-[2,6-dichloro-4-(trifluoro­meth­yl)phen­yl]-5-(4-methoxy­benzyl­ideneamino)-1*H*-pyrazole-3-carbonitrile

**DOI:** 10.1107/S1600536808005539

**Published:** 2008-02-29

**Authors:** Guohui Zhu, Shuyan Li, Zhiping Yang

**Affiliations:** a Department of Food and Biotechnology, Zhangzhou Vocational Technical College, 363000 Zhangzhou, People’s Republic of China

## Abstract

The title compound, C_19_H_10_BrCl_2_F_3_N_4_O, is an imine with an overall Y shape. The dihedral angles between the pyrazole ring and the methoxy- and trifluoromethyl-substituted benzene ring planes are 88.4 (2) and 65.8 (2)°, respectively.

## Related literature

For the insecticidal properties of similar compounds, see: Philippe (1997 ▶, 2000 ▶). For a related structure, see: Zhong *et al.* (2005 ▶).
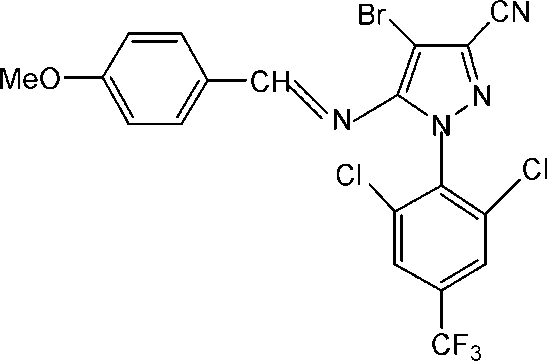


## Experimental

### 

#### Crystal data

C_19_H_10_BrCl_2_F_3_N_4_O
*M* *_r_* = 518.12
Monoclinic, 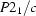

*a* = 10.215 (7) Å
*b* = 13.407 (9) Å
*c* = 15.108 (11) Å
β = 94.634 (13)°
*V* = 2062 (2) Å^3^
*Z* = 4
Mo *K*α radiation
μ = 2.30 mm^−1^
*T* = 293 (2) K
0.28 × 0.25 × 0.23 mm

#### Data collection

Bruker P4/SMART CCD diffractometer
Absorption correction: multi-scan (*SADABS*; Bruker, 2002 ▶) *T*_min_ = 0.568, *T*_max_ = 0.618
15375 measured reflections
4681 independent reflections
4061 reflections with *I* > 2σ(*I*)
*R*_int_ = 0.028

#### Refinement

*R*[*F* ^2^ > 2σ(*F* ^2^)] = 0.049
*wR*(*F* ^2^) = 0.160
*S* = 1.02
4681 reflections
271 parameters
H-atom parameters constrained
Δρ_max_ = 0.87 e Å^−3^
Δρ_min_ = −1.22 e Å^−3^

### 

Data collection: *SMART* (Bruker, 2002 ▶); cell refinement: *SAINT* (Bruker, 2002 ▶); data reduction: *SAINT*; program(s) used to solve structure: *SHELXS97* (Sheldrick, 2008 ▶); program(s) used to refine structure: *SHELXL97* (Sheldrick, 2008 ▶); molecular graphics: *ORTEP-3* (Farrugia, 1997 ▶); software used to prepare material for publication: *SHELXL97*.

## Supplementary Material

Crystal structure: contains datablocks global, I. DOI: 10.1107/S1600536808005539/sj2465sup1.cif

Structure factors: contains datablocks I. DOI: 10.1107/S1600536808005539/sj2465Isup2.hkl

Additional supplementary materials:  crystallographic information; 3D view; checkCIF report

## Figures and Tables

**Fig. 1.**
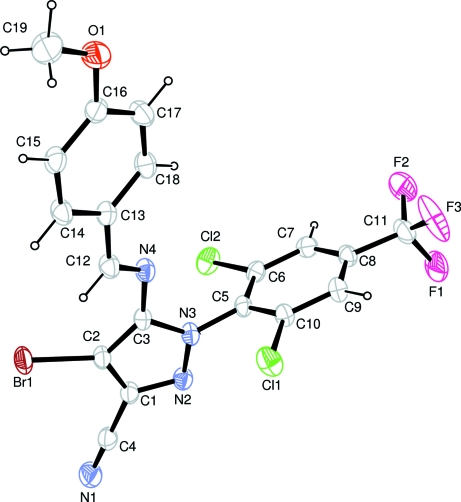
The molecular structure of (I) showing the atom numbering scheme and displacement ellipsoids at the 30% probability level.

## supplementary crystallographic information

### Comment

The title compound, (I), (Fig. 1) is similar to the very effective insecticides used to treat animals such as cows and sheep (Philippe, 1997, 2000) and its structure is reported here, Fig 1. The molecule contains three essentially planar rings and the dihedral angles between the pyrazole ring (C1—C3, N2, N3) and the ring planes of the (C5—C7) and (C13—C17) benzene rings are 88.4 (2)° and 65.8 (2)°, respectively.

### Experimental

The title compound was prepared by literature methods (Zhong *et al.*, 2005, Philippe, 2000). Colourless single crystals suitable for X-ray analysis were obtained by slow evaporation of an anhydrous ethanol-acetone (2:1) solution of (I) (m.p. 435–437 K).

### Refinement

All H atoms were initially located in a difference Fourier map but were eventually placed in their geometrically idealized positions and constrained to ride on their parent atoms, with C—H = 0.93 Å, and with *U*_iso_(H) = 1.2_eq_(C) and 0.96 Å, *U*_iso_ = 1.5*U*_eq_ (C) for CH_3_ atoms. The low *U*_eq_ of C11 as compared to its neighbours may be attributed to the high displacement parameters for atoms F1, F2 and F3, indicating either large thermal motion or rotational disorder of the trifluoromethyl group. However, attempts to represent the CF_3_ group using a disorder model were unsuccessful.

### Crystal data

**Table:**

C_19_H_10_BrCl_2_F_3_N_4_O	*F*_000_ = 1024
*M**_r_* = 518.12	*D*_x_ = 1.669 Mg m^−^^3^
Monoclinic, *P*2_1_/*c*	Melting point = 435–437 K
Hall symbol: -P 2ybc	Mo *K*α radiation λ = 0.71073 Å
*a* = 10.215 (7) Å	Cell parameters from 3780 reflections
*b* = 13.407 (9) Å	θ = 2.6–24.2º
*c* = 15.108 (11) Å	µ = 2.30 mm^−^^1^
β = 94.634 (13)º	*T* = 293 (2) K
*V* = 2062 (2) Å^3^	Block, colorless
*Z* = 4	0.28 × 0.25 × 0.23 mm

### Data collection

**Table:**

Bruker P4 CCD diffractometer	4681 independent reflections
Radiation source: fine-focus sealed tube	4061 reflections with *I* > 2σ(*I*)
Monochromator: graphite	*R*_int_ = 0.028
*T* = 293(2) K	θ_max_ = 27.6º
ω scans	θ_min_ = 2.8º
Absorption correction: multi-scan(SADABS; Bruker, 2002)	*h* = −13→10
*T*_min_ = 0.568, *T*_max_ = 0.618	*k* = −17→17
15375 measured reflections	*l* = −19→19

### Refinement

**Table:**

Refinement on *F*^2^	Secondary atom site location: difference Fourier map
Least-squares matrix: full	Hydrogen site location: inferred from neighbouring sites
*R*[*F*^2^ > 2σ(*F*^2^)] = 0.049	H-atom parameters constrained
*wR*(*F*^2^) = 0.160	*w* = 1/[σ^2^(*F*_o_^2^) + (0.1058*P*)^2^ + 1.2139*P*] where *P* = (*F*_o_^2^ + 2*F*_c_^2^)/3
*S* = 1.02	(Δ/σ)_max_ = 0.001
4681 reflections	Δρ_max_ = 0.87 e Å^−^^3^
271 parameters	Δρ_min_ = −1.22 e Å^−^^3^
Primary atom site location: structure-invariant direct methods	Extinction correction: none

### Special details

**Table:**

Geometry. All e.s.d.'s (except the e.s.d. in the dihedral angle between two l.s. planes) are estimated using the full covariance matrix. The cell e.s.d.'s are taken into account individually in the estimation of e.s.d.'s in distances, angles and torsion angles; correlations between e.s.d.'s in cell parameters are only used when they are defined by crystal symmetry. An approximate (isotropic) treatment of cell e.s.d.'s is used for estimating e.s.d.'s involving l.s. planes.
Refinement. Refinement of *F*^2^ against ALL reflections. The weighted *R*-factor *wR* and goodness of fit *S* are based on *F*^2^, conventional *R*-factors *R* are based on *F*, with *F* set to zero for negative *F*^2^. The threshold expression of *F*^2^ > σ(*F*^2^) is used only for calculating *R*-factors(gt) *etc*. and is not relevant to the choice of reflections for refinement. *R*-factors based on *F*^2^ are statistically about twice as large as those based on *F*, and *R*- factors based on All data will be even larger.

### Fractional atomic coordinates and isotropic or equivalent isotropic displacement parameters (Å^2^)

**Table:**

	*x*	*y*	*z*	*U*_iso_*/*U*_eq_	
Br1	0.47949 (4)	0.47599 (2)	0.18806 (3)	0.06204 (17)	
Cl1	0.47167 (9)	0.15617 (7)	−0.07867 (6)	0.0635 (3)	
Cl2	0.21780 (9)	0.09133 (6)	0.21206 (5)	0.0598 (2)	
O1	−0.1317 (3)	0.5592 (2)	−0.2291 (2)	0.0705 (7)	
N1	0.7437 (3)	0.2820 (3)	0.3107 (3)	0.0722 (9)	
N2	0.5089 (2)	0.17560 (18)	0.16494 (16)	0.0440 (5)	
N3	0.4063 (2)	0.19538 (16)	0.10558 (15)	0.0401 (5)	
N4	0.2718 (2)	0.32184 (17)	0.03993 (16)	0.0440 (5)	
C1	0.5457 (3)	0.2656 (2)	0.19544 (18)	0.0426 (6)	
C2	0.4669 (3)	0.34164 (19)	0.1561 (2)	0.0431 (6)	
C3	0.3773 (3)	0.29447 (18)	0.09722 (18)	0.0393 (5)	
C4	0.6555 (3)	0.2745 (2)	0.2597 (2)	0.0510 (7)	
C5	0.3380 (3)	0.11449 (18)	0.06234 (17)	0.0372 (5)	
C6	0.2496 (3)	0.0587 (2)	0.10637 (18)	0.0404 (5)	
C7	0.1868 (3)	−0.02304 (19)	0.0660 (2)	0.0455 (6)	
H7	0.1275	−0.0603	0.0959	0.055*	
C8	0.2134 (3)	−0.0482 (2)	−0.0186 (2)	0.0444 (6)	
C9	0.2998 (3)	0.0065 (2)	−0.0649 (2)	0.0467 (6)	
H9	0.3161	−0.0110	−0.1225	0.056*	
C10	0.3616 (3)	0.0880 (2)	−0.02374 (18)	0.0412 (5)	
C11	0.1513 (4)	−0.1404 (2)	−0.0601 (3)	0.0606 (8)	
C12	0.2714 (3)	0.4090 (2)	0.0053 (2)	0.0468 (6)	
H12	0.3433	0.4503	0.0194	0.056*	
C13	0.1648 (3)	0.4469 (2)	−0.05455 (19)	0.0423 (6)	
C14	0.1740 (3)	0.5440 (2)	−0.0862 (2)	0.0499 (7)	
H14	0.2473	0.5823	−0.0682	0.060*	
C15	0.0759 (3)	0.5846 (2)	−0.1438 (2)	0.0485 (6)	
H15	0.0826	0.6499	−0.1637	0.058*	
C16	−0.0316 (3)	0.5275 (2)	−0.1712 (2)	0.0474 (6)	
C17	−0.0425 (3)	0.4296 (3)	−0.1403 (2)	0.0556 (7)	
H17	−0.1157	0.3913	−0.1586	0.067*	
C18	0.0549 (3)	0.3903 (2)	−0.0829 (2)	0.0499 (7)	
H18	0.0476	0.3252	−0.0627	0.060*	
C19	−0.1250 (5)	0.6581 (3)	−0.2612 (4)	0.0878 (15)	
H19C	−0.2005	0.6714	−0.3015	0.132*	
H19B	−0.0467	0.6660	−0.2916	0.132*	
H19A	−0.1233	0.7039	−0.2123	0.132*	
F1	0.2269 (3)	−0.21908 (16)	−0.0449 (2)	0.0972 (10)	
F2	0.0391 (3)	−0.1633 (2)	−0.0287 (3)	0.1064 (11)	
F3	0.1310 (4)	−0.1354 (3)	−0.1460 (2)	0.1269 (15)	

### Atomic displacement parameters (Å^2^)

**Table:**

	*U*^11^	*U*^22^	*U*^33^	*U*^12^	*U*^13^	*U*^23^
Br1	0.0722 (3)	0.0340 (2)	0.0776 (3)	−0.00455 (12)	−0.00746 (19)	−0.01343 (13)
Cl1	0.0740 (5)	0.0690 (5)	0.0497 (4)	−0.0276 (4)	0.0184 (4)	−0.0037 (3)
Cl2	0.0782 (5)	0.0583 (5)	0.0453 (4)	0.0063 (4)	0.0197 (4)	0.0037 (3)
O1	0.0646 (15)	0.0602 (15)	0.0822 (18)	0.0061 (12)	−0.0225 (13)	0.0004 (13)
N1	0.0669 (19)	0.0670 (19)	0.079 (2)	0.0077 (15)	−0.0188 (17)	−0.0201 (16)
N2	0.0514 (13)	0.0380 (12)	0.0416 (11)	0.0037 (10)	−0.0028 (10)	−0.0017 (9)
N3	0.0520 (12)	0.0308 (10)	0.0366 (10)	−0.0008 (9)	−0.0025 (9)	−0.0019 (8)
N4	0.0547 (13)	0.0327 (11)	0.0434 (12)	0.0007 (9)	−0.0037 (10)	−0.0006 (9)
C1	0.0483 (14)	0.0377 (13)	0.0412 (13)	0.0013 (10)	0.0008 (11)	−0.0060 (10)
C2	0.0517 (15)	0.0309 (12)	0.0460 (14)	−0.0012 (10)	−0.0002 (11)	−0.0049 (10)
C3	0.0478 (13)	0.0294 (11)	0.0407 (13)	−0.0014 (10)	0.0045 (10)	−0.0017 (9)
C4	0.0555 (17)	0.0464 (15)	0.0501 (16)	0.0048 (12)	−0.0012 (13)	−0.0126 (12)
C5	0.0458 (13)	0.0271 (11)	0.0381 (12)	0.0005 (9)	0.0002 (10)	−0.0006 (9)
C6	0.0481 (14)	0.0325 (12)	0.0410 (13)	0.0050 (10)	0.0052 (11)	0.0046 (10)
C7	0.0440 (14)	0.0331 (13)	0.0596 (17)	−0.0016 (10)	0.0057 (12)	0.0084 (11)
C8	0.0461 (14)	0.0305 (12)	0.0551 (16)	−0.0028 (10)	−0.0045 (12)	−0.0017 (11)
C9	0.0571 (16)	0.0409 (13)	0.0419 (14)	−0.0053 (12)	0.0019 (12)	−0.0070 (11)
C10	0.0477 (14)	0.0372 (13)	0.0392 (13)	−0.0059 (10)	0.0053 (10)	−0.0023 (10)
C11	0.0627 (19)	0.0413 (16)	0.075 (2)	−0.0116 (14)	−0.0131 (16)	−0.0049 (15)
C12	0.0539 (16)	0.0358 (13)	0.0498 (15)	−0.0028 (11)	−0.0012 (12)	0.0000 (11)
C13	0.0527 (15)	0.0331 (12)	0.0407 (13)	−0.0004 (11)	0.0027 (11)	−0.0002 (10)
C14	0.0570 (17)	0.0344 (13)	0.0567 (17)	−0.0059 (12)	−0.0056 (13)	0.0023 (12)
C15	0.0604 (17)	0.0320 (12)	0.0522 (16)	0.0032 (11)	−0.0007 (13)	0.0019 (11)
C16	0.0513 (16)	0.0440 (16)	0.0461 (15)	0.0069 (11)	−0.0005 (12)	−0.0038 (11)
C17	0.0546 (17)	0.0481 (17)	0.0628 (19)	−0.0122 (13)	−0.0031 (14)	−0.0011 (14)
C18	0.0614 (17)	0.0363 (13)	0.0522 (16)	−0.0082 (12)	0.0061 (13)	0.0024 (11)
C19	0.091 (3)	0.059 (2)	0.106 (4)	0.020 (2)	−0.035 (3)	0.006 (2)
F1	0.0977 (18)	0.0389 (11)	0.150 (3)	0.0004 (11)	−0.0223 (18)	−0.0259 (14)
F2	0.0762 (16)	0.0812 (18)	0.164 (3)	−0.0395 (14)	0.0210 (18)	−0.0324 (19)
F3	0.198 (4)	0.095 (2)	0.0784 (18)	−0.074 (2)	−0.044 (2)	−0.0037 (16)

### Geometric parameters (Å, °)

**Table:**

Br1—C2	1.867 (3)	C8—C11	1.503 (4)
Cl1—C10	1.714 (3)	C9—C10	1.384 (4)
Cl2—C6	1.712 (3)	C9—H9	0.9300
O1—C16	1.359 (4)	C11—F3	1.299 (5)
O1—C19	1.416 (5)	C11—F2	1.311 (5)
N1—C4	1.142 (5)	C11—F1	1.317 (4)
N2—C1	1.334 (4)	C12—C13	1.450 (4)
N2—N3	1.349 (3)	C12—H12	0.9300
N3—C3	1.365 (3)	C13—C18	1.393 (4)
N3—C5	1.420 (3)	C13—C14	1.393 (4)
N4—C12	1.280 (4)	C14—C15	1.385 (4)
N4—C3	1.376 (4)	C14—H14	0.9300
C1—C2	1.402 (4)	C15—C16	1.374 (5)
C1—C4	1.428 (4)	C15—H15	0.9300
C2—C3	1.377 (4)	C16—C17	1.401 (5)
C5—C6	1.383 (4)	C17—C18	1.370 (5)
C5—C10	1.388 (4)	C17—H17	0.9300
C6—C7	1.386 (4)	C18—H18	0.9300
C7—C8	1.371 (5)	C19—H19C	0.9600
C7—H7	0.9300	C19—H19B	0.9600
C8—C9	1.380 (4)	C19—H19A	0.9600
			
C16—O1—C19	117.3 (3)	F3—C11—F2	107.5 (3)
C1—N2—N3	103.5 (2)	F3—C11—F1	105.0 (4)
N2—N3—C3	113.8 (2)	F2—C11—F1	105.5 (3)
N2—N3—C5	118.8 (2)	F3—C11—C8	113.7 (3)
C3—N3—C5	127.3 (2)	F2—C11—C8	113.2 (3)
C12—N4—C3	118.5 (3)	F1—C11—C8	111.4 (3)
N2—C1—C2	112.0 (2)	N4—C12—C13	123.5 (3)
N2—C1—C4	119.7 (3)	N4—C12—H12	118.3
C2—C1—C4	128.3 (3)	C13—C12—H12	118.3
C3—C2—C1	105.6 (2)	C18—C13—C14	118.6 (3)
C3—C2—Br1	129.7 (2)	C18—C13—C12	123.2 (3)
C1—C2—Br1	124.6 (2)	C14—C13—C12	118.2 (3)
N3—C3—N4	118.1 (2)	C15—C14—C13	121.1 (3)
N3—C3—C2	105.0 (2)	C15—C14—H14	119.4
N4—C3—C2	136.8 (2)	C13—C14—H14	119.4
N1—C4—C1	179.6 (5)	C16—C15—C14	119.4 (3)
C6—C5—C10	118.8 (2)	C16—C15—H15	120.3
C6—C5—N3	120.5 (2)	C14—C15—H15	120.3
C10—C5—N3	120.7 (2)	O1—C16—C15	124.1 (3)
C5—C6—C7	120.8 (3)	O1—C16—C17	115.5 (3)
C5—C6—Cl2	119.4 (2)	C15—C16—C17	120.3 (3)
C7—C6—Cl2	119.8 (2)	C18—C17—C16	119.8 (3)
C8—C7—C6	119.1 (3)	C18—C17—H17	120.1
C8—C7—H7	120.5	C16—C17—H17	120.1
C6—C7—H7	120.5	C17—C18—C13	120.8 (3)
C7—C8—C9	121.6 (3)	C17—C18—H18	119.6
C7—C8—C11	118.9 (3)	C13—C18—H18	119.6
C9—C8—C11	119.5 (3)	O1—C19—H19C	109.5
C8—C9—C10	118.7 (3)	O1—C19—H19B	109.5
C8—C9—H9	120.7	H19C—C19—H19B	109.5
C10—C9—H9	120.7	O1—C19—H19A	109.5
C9—C10—C5	121.0 (2)	H19C—C19—H19A	109.5
C9—C10—Cl1	119.8 (2)	H19B—C19—H19A	109.5
C5—C10—Cl1	119.2 (2)		
